# Iron toxicity undermines microfracture-induced cartilage regeneration by predisposing a pre-ferroptotic niche

**DOI:** 10.3389/fcell.2026.1784707

**Published:** 2026-03-11

**Authors:** Haining Peng, Zhongkai Ren, Yingze Zhang, Tengbo Yu, Xiaohong Huang

**Affiliations:** 1 Department of Orthopedics, The Affiliated Hospital of Qingdao University, Qingdao University, Qingdao, China; 2 Department of Orthopedic Surgery, Qingdao Hospital, University of Health and Rehabilitation Sciences (Qingdao Municipal Hospital), Qingdao, China; 3 Shandong Institute of Traumatic Orthopedics, Medical Research Center, The Affiliated Hospital of Qingdao University, Qingdao, China; 4 Department of Orthopedics, The Third Hospital of Hebei Medical University, Shijiazhuang, China

**Keywords:** cartilage regeneration, ferroptosis, iron, microfracture, sphingolipid, transferrin

## Abstract

Microfracture (MF) often yields regenerated cartilage that resembles scar tissue and is prone to rapid deterioration. This outcome may be linked to elevated iron levels, which upregulate sphingolipid (SP) signaling and increase lipid exposure to reactive oxygen species (ROS), thereby heightening cellular sensitivity to iron. In this study, we analyze whether heme-derived iron released during clinical MF undermines cartilage regeneration. We compared regenerated and intact cartilage using histomorphological, proteomic, metabolomic, and transcriptional analyses. Regenerated tissue exhibited disrupted cellular organization and a deficient extracellular matrix. Omics profiling highlighted transferrin-mediated iron transfer, striking SP signaling, and increased oxidized glutathione tripeptide in cartilage regeneration. Integrated analysis further revealed a pre-ferroptotic microenvironment in newborn chondrocytes after MF, which is characterized by extracellular Fe^3+^ accumulation, moderately increased Fe^2+^ levels, heterogeneous expression of ferroptotic markers, and altered mitochondrial and lysosomal structures. To assess the role of iron toxicity and iron-dependent oxidative stress, we administered intra-articular injections of the iron chelator deferoxamine (DFO) or the lipid ROS scavenger ferrostatin-1 (FER-1). Both treatments improved joint mobility, increased regenerated tissue thickness, elevated proteoglycan content, reduced sphingomyelin levels, preserved mitochondrial structure, and decreased lysosome abundance. These findings demonstrate that iron toxicity establishes a pre-ferroptotic niche that compromises cartilage regeneration following MF. In this study, we provide new mechanistic insights for developing targeted therapeutic strategies to enhance cartilage restoration.

## 1 Introduction

Cartilage, an avascular supporting connective tissue, presents a significant challenge for regeneration. Due to its inherently limited healing capacity, the progressive wear of this protective layer between bones leads to osteoarthritis (OA) (Li et al., 2022; Xu et al., 2023). In clinical practice, arthroscopic microfracture (MF) is a widely adopted surgical strategy to promote cartilage repair (Devitt et al., 2017). This procedure involves creating small perforations in the subchondral bone, facilitating the influx of bone marrow-derived mesenchymal stem cells (BMSCs) via the bloodstream to the site of impairment (Gomoll, 2012; Murphy et al., 2020). However, the resultant regenerated tissue often constitutes a fibrotic scar, which lacks the biomechanical properties of natural hyaline cartilage. This deficiency is primarily attributed to the insufficient deposition of key extracellular matrix components, notably, aggrecan (Acan) and type II collagen (Col2a1), rendering the repair tissue prone to rapid deterioration (Valiyaveettil et al., 2005; Armiento et al., 2019). The precise molecular mechanisms underlying these suboptimal clinical outcomes following MF remain incompletely understood.

Iron accumulation occurs in organisms during aging (Ru et al., 2023). Iron exerts cellular toxicity primarily by inducing the generation of reactive oxygen species (ROS), which exposes macromolecules such as lipids to oxidative damage (Isidori et al., 2018). As a protective mechanism, organelles such as lysosomes sequester iron, thereby reducing the exposure of cytosolic and nuclear macromolecules to elevated iron levels (Pamarthi et al., 2022). However, increased ROS-mediated lysosomal permeabilization can trigger ferroptosis in cancer contexts (Chen et al., 2023). A high-iron status upregulates sphingolipid (SP) signaling, and SPs subsequently heighten cellular sensitivity to iron toxicity (Lee et al., 2012). The intracellular accumulation of iron initiates ferroptosis (Ajoolabady et al., 2021), a process exacerbated by ceramide (Cer) produced from the degradation of sphingomyelins (SMs, a subclass of SPs) (Thayyullathil et al., 2021). Ferroptosis is implicated in the pathogenesis of various orthopedic disorders (Miao et al., 2022; Ru et al., 2023). In OA, elevated iron concentrations and lipid peroxidation are observed in damaged cartilage, and a distinct subpopulation of ferroptotic chondrocytes has been identified (Lv et al., 2022). These findings indicate that mitigating iron toxicity may represent a promising strategy to preserve cartilage function and prevent its degradation.

We hypothesize that heme-derived iron, released during the clinical MF procedure, impairs cartilage regeneration by exerting toxic effects on newborn chondrocytes at the defect site. In this study, we characterized the histomorphological, proteomic, and metabolomic differences between regenerated and intact cartilage. We further assessed iron-predisposed chondrotoxicity by analyzing alterations in cartilage iron deposition and ferroptotic marker expression and evaluated its rescue through the chelation of excess iron or the inhibition of iron-dependent oxidative stress.

## 2 Materials and methods

### 2.1 Ethics statement

This study was approved by the Ethics Committee for Experimental Animals of the Affiliated Hospital of Qingdao University (QYFY WZLL 28062) and conducted in accordance with the ARRIVE guidelines.

### 2.2 Animals and sample preparation

A total of 90 New Zealand white rabbits (male, 10–12 weeks, 2.0 kg–2.5 kg) were used. Specifically, 15 rabbits were subjected to MF and 15 to control (CON) to explore the molecular mechanisms underlying insufficient cartilage regeneration; a further 60 rabbits were subjected to MF to determine the effectiveness of deferoxamine (DFO) and ferrostatin-1 (FER-1) in improving MF outcomes. All rabbits were sacrificed by CO_2_ asphyxiation at 4 weeks after surgery.

For omics (n = 3 for proteomics and n = 6 for lipidomics), the cartilage samples were dissected from distal femurs, washed with phosphate-buffered saline (PBS), and stored at −80 °C. For histological and immunohistochemical (IHC) analyses (n = 3), the isolated distal femur trochlear grooves were kept in 4% paraformaldehyde in PBS (PFA) for 24 h and decalcified in ethylenediaminetetraacetic acid (EDTA, 14% w/v, pH 7.4) for 21 days at 4 °C. For transmission electron microscope (TEM) observation (n = 3), the cartilage tissues were cut into 1 mm^3^ sections and fixed in a fixative solution (G1102, ServiceBio, China). To quantify the Fe^2+^ content in the cartilage (n = 3), the isolated cartilage was homogenized with cold saline (10% w/v). For real-time PCR (n = 3), the cartilage was washed and stored at −80 °C.

### 2.3 Microfracture model

The rabbits were anesthetized through ear vein administration of 3% w/v pentobarbital sodium at a dosage of 1.0 mg/kg. The MF surgery was conducted on the left femur as described in our previous report (Peng et al., 2022). A defect in the articular cartilage (Ø ∼ 4 × 6 mm, 2 mm in depth) was created in the trochlear groove of the left distal femur. The cartilage from the left femurs of the CON rabbits was exposed without drilling holes. The patella was repositioned, and the wound was sutured. The rabbits were treated with gentamycin for 3 days after surgery.

### 2.4 Drugs and dosages

DFO (GC13554, GlpBio, United States), FER-1 (GC10380, GlpBio), dimethyl sulfoxide (DMSO, D8371, Solarbio, China), polyethylene glycol (PEG, IP9020, Solarbio), Tween-80 (T8360, Solarbio), and saline (B1219, APPLYGEN, China) were purchased. The vehicle contained 10% DMSO, 40% PEG, 5% Tween-80, and 45% saline. FER-1 (a lipid ROS scavenger, 0.5 mg/kg), DFO (an iron chelator, 100 mg/kg), or an equivalent volume of vehicle was intra-articularly injected in the MF animals on days 5, 12, 19, and 26 post-surgery.

### 2.5 Gait analysis

The gait analysis was conducted on days 14, 21, and 28 post-surgery. Each foot of the rabbits was pigmented with non-toxic, washable ink. Tracks were formed when the rabbits walked on the sheet. The length of stride of the limbs was measured for the left forelimb (LF), right forelimb (RF), left hindlimb (LH), and right hindlimb (RH), along with the width of stride between the hindlimbs (width).

### 2.6 Proteomics

Tandem mass tag (TMT)-based quantitative proteomics was performed on intact and regenerated samples as described previously (Liu et al., 2023). In brief, the proteins were extracted using the phenol extraction method, and the protein concentration was determined using the bicinchoninic acid (BCA) kit (Thermo Fisher Scientific, United States). Then, 50 μg of proteins were hydrolyzed with trypsin and labeled with a TMT reagent. The TMT-labeled peptides were separated by high-pH reversed-phase liquid chromatography using a Thermo Dionex UltiMate 3000 RSLCnano System (Thermo Fisher Scientific). The peptide sequence was determined using a Q-Exactive HF mass spectrometer (Thermo Fisher Scientific).

The peptides were identified by searching among all the reviewed rabbit entries from UniProt (downloaded on 15 April 2022) using Proteome Discoverer (v2.4, Thermo Fisher Scientific). To ensure the comparability of protein abundance across samples, raw reporter ion intensities were log_2_-transformed and normalized using the median centering method. This normalization procedure effectively corrected for technical variations in TMT labeling efficiency and sample loading. Principal component analysis (PCA) was utilized to distinguish the proteomic profiles between the CON and MF groups, and a volcano plot was used to visualize the changes in the protein expression in cartilage regeneration. To highlight the kill switch in the sufficient cartilage regeneration, a threshold to identify the differentially expressed proteins (DEPs) was set at |Log_2_FC| ≥ 1 and 2 with a *p*-value <0.05. Then, Gene Ontology (GO) enrichment analysis based on the Gene ID of the DEPs was performed using R based on the hypergeometric distribution. The pathway enrichment analysis for the DEPs was performed using the Kyoto Encyclopedia of Genes and Genomes database (KEGG) using the matching IDs. A path with a *p*-value <0.05 was considered significant.

### 2.7 Metabolomic analysis

Raw data from our previously reported comparative metabolomics were utilized (Peng et al., 2022). Metabolites were identified using the Human Metabolome Database (HMDB), Lipid Maps (v2.3), METLIN software, and a self-built database of Shanghai Lu-Ming Biotech Co., Ltd. (Shanghai, China). For the differentially expressed metabolites (DEMs), a threshold value was set at *p*-value <0.05 and variable influence on projection (VIP) > 1. DEMs were then mapped to the KEGG database to illustrate deficiencies in functional pathways associated with insufficient cartilage regeneration. Specifically, the DEMs were sorted based on the PubChem classification (https://pubchem.ncbi.nlm.nih.gov/) to gain insight into the primary deficiency of insufficient cartilage regeneration.

### 2.8 Integrated omics analysis

The DEPs (*p*-value <0.05, |Log_2_FC| ≥ 1) and DEMs (*p*-values <0.05, VIP >1) were utilized to explore the protein–metabolite integrated trajectories in a regenerative process. KEGG Markup Language (KGML) network analysis was launched to identify the primary signaling pathways accounting for the insufficient cartilage regeneration. To specify the relationship between ferroptosis and insufficient cartilage regeneration, 621 ferroptosis-related genes were downloaded from FerrDb (http://www.zhounan.org/ferrdb/) and intersected with the DEPs.

### 2.9 Lipidomics

Untargeted lipidomic analysis was carried out as described (Zhang et al., 2021). In brief, the metabolic profile was analyzed using an ultra-performance liquid chromatography (UPLC) system (Nexera, Shimadzu Corporation, Japan) coupled with Q-Exactive plus quadrupole-Orbitrap MS (Thermo Fisher Scientific). The raw scans were performed with LipidSearch (v4.2, Thermo Fisher Scientific). In addition, the lipid identification, i.e., the molecular structure of the lipids and the additive mode of its positive and negative ions, was determined based on the parent ions and multi-stage MS data. The results were aligned according to a specified retention time range and combined into a single report to organize the original data matrix.

The supervised OPLS–DA analysis was used to distinguish the lipidomic profiles of the intervention (DFO or FER-1) from those of the vehicle. A volcano plot was used to visualize the lipidomic changes induced by the intervention. For screening DEMs, a threshold was set at a *p*-value <0.05 and VIP >1. KEGG enrichment was performed, and the signaling pathways were considered significant with a *p*-value <0.05.

### 2.10 Histological analyses

The samples were embedded in paraffin, and the sagittal sections were cut at a thickness of 3 μm. Hematoxylin and eosin (H&E) and safranin-O/fast green (SO/FG) staining were conducted to detect the morphological alteration and proteoglycan content in the cartilage. To analyze Fe^3+^ deposition, the slides were stained using a Prussian blue stain kit (G1428, Solarbio). The slices were dehydrated, transparentized, and mounted with neutral resin for microscopic observation (Ni-U, Nikon, Japan). The images were representative of the results of three biological replicates.

### 2.11 Immunohistochemistry

The IHC analysis was conducted using an SP Rabbit Kit (SP0021, Solarbio). The slides were heated at 37 °C for 30 min, and the sections were treated with 3% H_2_O_2_ in the dark at room temperature for 25 min. Nonspecific binding sites were blocked by 10% bovine serum albumin (BSA) for 30 min at room temperature. The sections were incubated with the primary antibodies overnight at 4 °C, respectively, including Acan (1:500, bs-1223R, Boiss, China), Col2a1 (1:500, bs-10589R, Boiss), Col1a1 (1:500, bs-10423R, Boiss), matrix metalloproteinase 3 (Mmp3, 1:500, bs-0413R, Boiss), transferrin (Tf, 1:500, bs-0988R, Boiss), transferrin receptor (Tfrc, 1:500, bs-0988R, Boiss), heme-oxygenase 1 (Hmox1, 1:500, bs-2075R, Boiss), matrix metalloproteinase 13 (Mmp13, 1:500, bs-0575R, Boiss), and heme-oxygenase 2 (Hmox2, 1:300, 14817-1-AP, Proteintech, China). The sections were incubated with the biotinylated secondary antibody, counterstained with hematoxylin, visualized using diaminobenzidine (DAB) solution, and mounted with neutral resin. The slides were observed under a bright-field microscope (Ni-U, Nikon, Japan).

### 2.12 Transmission electron microscopy

The fixed tissue was washed and post-fixed with 1% aqueous osmium tetroxide (OsO_4_, Ted Pella, Inc., United States) while protected from light, followed by dehydration in a graded ethanol series (from 30% to 100%) and acetone (100%). The samples were prepared by gradient infiltration of anhydrous acetone and EMBed 812 (90529-77-4, Structure Probe, Inc., United States) overnight, embedded in EMBed 812, and polymerized at 60 °C for 48 h. The embedded tissue was cut into 80-nm sections using an ultra-microtome (Leica UC7, Leica, Herlev, Denmark). The section was mounted onto 150-mesh copper grids with formvar film, stained with 2% uranium acetate and 2.6% lead citrate, and observed using TEM (HT7800, HITACHI, Japan).

### 2.13 Iron measurement

A tissue iron assay kit (A039-two to one, Nanjing Jian Cheng Bioengineering Institute, China) was used to determine the Fe^2+^ content in the cartilage. The absorbance value of each sample was determined by averaging the reads from two technical replicates using a multimode plate reader (PerkinElmer, United States) at 520 nm.

### 2.14 Real-time PCR

The total RNA was extracted using NcmZol Reagent (M5100, NCM Biotech, China) and quantified using a spectrophotometer (NanoDrop 2000c; Thermo Fisher Scientific). Reverse transcription was performed using an Evo M-MLV RT Mix kit with gDNA Clean for qPCR (AG11728, Accurate Biology, China). Real-time PCR was carried out using a SYBR Green Pro Taq HS qPCR Kit (AG11701, Accurate Biology) and gene-specific primers (Table 1) on QuantStudio 5 (Applied Biosystems, United States). β-actin was used as the internal control. The relative mRNA expressions were calculated using the 2^−ΔΔCT^ method.

**TABLE 1:** List of the primers used in the real-time PCR analysis.

Gene	Primer
β-actin	F: TGG​CTC​TAA​CAG​TCC​GCC​TAG
	R: AGT​GCG​ACG​TGG​ACA​TCC​G
Acan	F: ATC​TGG​AGT​TCT​TTT​TGG​GA
	R: AGG​TCA​GGG​ATT​CTG​TGT​GT
Col2a1	F: CAA​TGG​CGG​CTT​CCA​CTT​CAG
	R: GAC​AGT​CTT​GCC​CCA​CTT​ACC
Col1a1	F: CCC​ACT​GGT​GTT​CAA​GGA​CC
	R: TTC​CAG​TGA​GAC​CCT​TGG​CA
Mmp3	F: TTA​GAG​GAA​ATG​AGG​TAC​AAG​C
	R: ATC​CAC​TGA​AGA​AAT​AGA​AAA​A
Mmp13	F: CTG​CCC​CTC​CTC​AAC​AGT​AA
	R: CCT​GTC​ACC​TCT​AAG​CCG​AA
Tf	F: GAA​GGG​TAT​CTT​TCT​GTG​GC
	R: CAG​TGG​TTG​ATC​CTG​TTG​TA
Tfrc	F: AAA​ATG​CAG​AAA​TAA​CCA​ACA
	R: TCC​CTG​AAT​AGT​CCA​AGT​AGC
Hmox1	F: CAG​GCA​GAG​GGT​GGT​GGA​AGA
	R: GAA​GGA​GCG​GAG​GCT​GGG​AGA
Hmox2	F: ACC​ACC​GCA​CTT​TAC​TTC​ACA
	R: CCT​GCT​CCT​CCC​AGT​TCT​CAC

### 2.15 Statistics

Data analysis was performed using Prism (v9.0, United States). Data were presented as the mean ± standard error (SE). Student’s *t*-test was used to determine the difference between two groups. Moreover, the effects of intervention, time, and their interaction were revealed by a two-way ANOVA; the Tukey–Kramer test was used to partition differences between treatments at each time point. A value of *p* < 0.05 was considered statistically significant.

## 3 Results

### 3.1 Insufficient extracellular matrix in the regenerated cartilage

**FIGURE 1:**
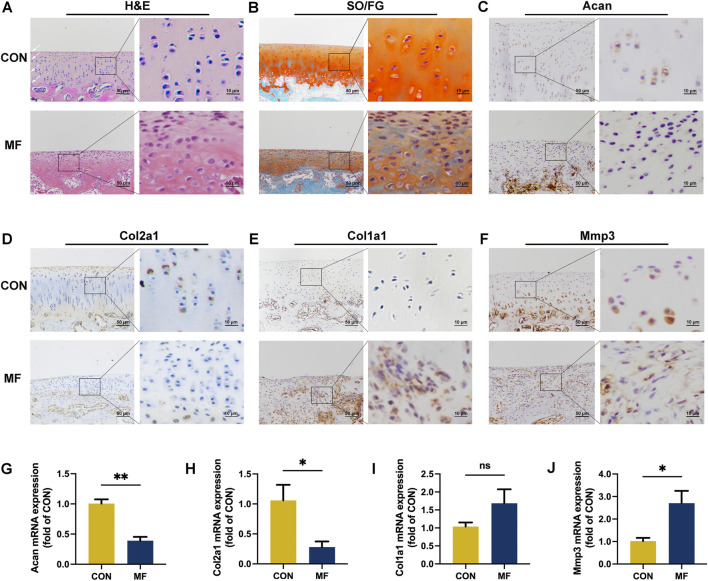
Insufficient cartilage regeneration is the main outcome of MF. **(A)** H&E staining of the normal cartilage and regenerated tissue. **(B)** SO/FG staining. **(C–F)** Immunohistochemical staining of Acan, Col2a1, Col1a1, and Mmp3. The images are the representative result of three biological replicates. **(G–J)** Relative mRNA expressions of Acan, Col2a1, Col1a1, and Mmp3. The data are presented as the mean ± SE (n = 3). ns indicates no significance (*p* > 0.05); *p < 0.05; **p < 0.01.

The intact cartilage exhibited a highly organized four-layer structure, while the regenerated tissue lost its cellular regularity (Figure 1A). Tissue thickness decreased while chondrocyte density increased at the site of the original impairment (Figure 1A; Supplementary Figure S1), indicating altered ECM structure and chondrocyte dysfunction in cartilage regeneration. Proteoglycans, the vital components of ECM, account for the elastic property of cartilage (Kim et al., 2015). It has been found that the content of proteoglycans in the regenerated tissue was decreased, as indicated by a lighter reddish staining compared to that of the normal cartilage (Figure 1B).

The positive staining of Acan, a major proteoglycan in cartilage (Kim et al., 2015), was mainly detected in the middle, deep, and calcified layers of normal cartilage, and it was mostly absent in MF-induced cartilage (Figure 1C). The mRNA expression of Acan was reduced to less than half of that in the normal (Figure 1G). Col2a1 is a hyaline cartilage chondrocyte marker, while collagen I (Col1a1) is the fibrocartilage chondrocyte marker and is highly expressed in the primary OA chondrocytes (Ripmeester et al., 2021). In the intact cartilage, Col2a1 was evenly distributed, and the distribution pattern of MF-induced Col2a1 was similar to that of Acan (Figure 1D). The mRNA expression of Col2a1 was decreased in the MF group compared to that in CON (Figure 1H). On the contrary, a small Col1a1 staining was detected in the calcified layer in the undamaged cartilage, whereas increased Col1a1-positive staining under the superficial layer was observed in the MF group (Figure 1E). In addition, the mRNA expression of MF-induced Col1a1 increased by three-quarters of the level in the CON group, yet statistical significance was not achieved (Figure 1I). Mmp3 digests ECM components, activates the pro-forms of matrix metalloproteinases, and contributes to the activation of aggrecanase II (Leong et al., 2010). In the CON group, the Mmp3-positive cells were detected in the deep and calcified layers (Figure 1F), while Mmp3 expression increased 1.5-fold in the MF group compared to that in the CON group (Figure 1J), which is consistent with the rapid degradation of the regenerated tissue (Armiento et al., 2019).

### 3.2 Abnormal iron metabolism in the regenerated cartilage

**FIGURE 2:**
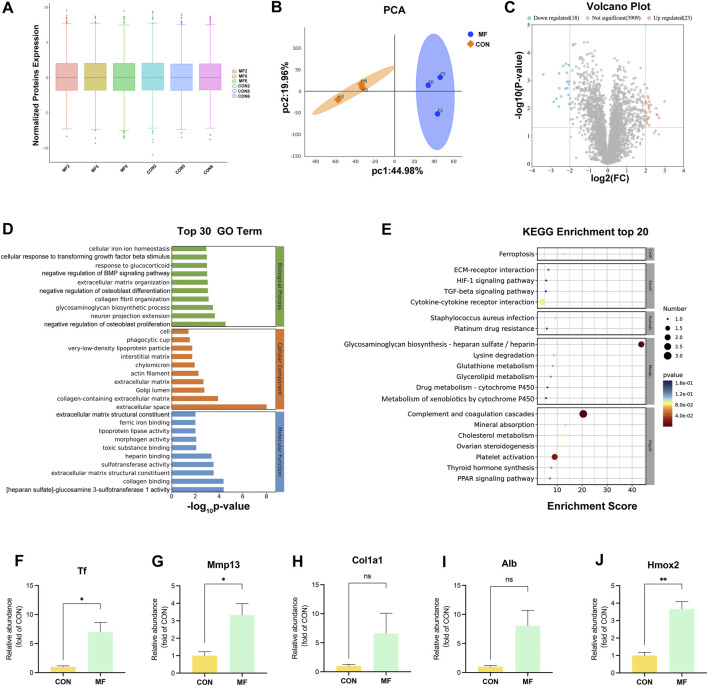
Proteomic profile alterations in MF-induced cartilage regeneration. **(A)** Three samples from the MF group and three samples from the CON group are subjected to proteomic analysis. **(B)** PCA indicates the distinct difference between the CON and MF groups. **(C)** The volcano plot reveals the DEPs at a threshold of |Log2FC| ≥ 2 and *p*-value <0.05. **(D)** Top 30 GO terms enriched by the DEPs (Supplementary Table S2). **(E)** Top 20 KEGG pathways enriched by the DEPs (Supplementary Table S3). The bubble size represents the number of proteins detected in the KEGG pathway, and the color of the bubble represents the *p*-value of the pathway. **(F–J)** Relative abundance of Tf, Mmp13, Alb, Col1a1, and Hmox2 are revealed between the CON and MF groups. The data are presented as the mean ± SE (n = 3). ns indicates no significance (*p* > 0.05); *p < 0.05; **p < 0.01.

Comparative proteomics was carried out to analyze the molecular mechanism underlying abnormal ECM constitution and chondrocyte dysfunction in the regenerated tissue (Figure 2A). A distinct difference between the MF and CON groups was revealed (Figure 2B). A total of 325 DEPs was screened (*p* < 0.05, |Log_2_ FC| ≥ 1; Supplementary Table S1), and the alteration in the SP signaling pathway was revealed by KEGG enrichment (Supplementary Figure S2). To highlight critical regulators in the regenerative process, the threshold was set to *p* < 0.05 and |Log_2_ FC| ≥ 2, resulting in a total of 41 DEPs (Table 2; Figure 2C). GO annotation revealed ECM-related terms such as extracellular space, collagen-containing ECM, collagen binding, cellular iron homeostasis, and Fe^3+^ binding (Figure 2D; Supplementary Table S2). At the same time, signaling pathways involved in ferroptosis and ECM-receptor interaction were enriched by KEGG analysis (Figure 2E; Supplementary Table S3).

**TABLE 2:** List of the differentially expressed proteins between the normal and regenerated cartilages.

Accession	Protein name	Gene symbol	Log_2_(FC)	*p*-value
Q6GVI2	Prepro-alpha-1 collagen type I (fragment)	​	2.99	0.01
G1TKR0	Tenascin N	TNN	2.70	0.02
A0A5F9DEY4	Kinesin family member 21A	KIF21A	2.62	0.02
A0A5F9D9X3	Periostin	POSTN	2.58	0.02
G1SIV6	Pannexin	PANX3	2.55	0.01
G1U9S2	Albumin	ALB	2.28	0.01
A0A5F9DM51	Uncharacterized protein	​	2.21	0.01
A0A5F9CJC8	Periostin	POSTN	2.18	0.01
G1TVI8	Sterile alpha motif domain containing 9 like	SAMD9L	2.18	0.04
Q9TTJ4	Inter-alpha-trypsin inhibitor heavy chain H1	ITI	2.17	0.01
G1SUI9	Uncharacterized protein	CALD1	2.17	0.01
A0A5F9DHV4	Fibrinogen gamma chain	FGG	2.17	0.02
A0A1Y1B8B8	IgG light chain (fragment)	​	2.16	0.01
P19134	Serotransferrin	TF	2.14	<0.01
G1SP04	Calmodulin-lysine N-methyltransferase	CAMKMT	2.11	<0.01
G1TK85	Immunoglobulin heavy constant mu	IGHM	2.09	0.02
G1T0X2	Fibrinogen alpha chain	FGA	2.08	0.02
G1TXD9	Allograft inflammatory factor 1	AIF1	2.06	0.04
A0A5F9DND9	Coagulation factor V	F5	2.06	<0.01
G1SP97	Lumican	LUM	2.05	0.03
G1U985	Stabilin 1	STAB1	2.04	0.01
A0A5F9CKC6	AE binding protein 1	AEBP1	2.03	0.01
G1T1T9	General transcription factor IIIA	GTF3A	2.03	0.02
A0A5F9DTX7	3Beta_HSD domain-containing protein	​	−2.04	<0.01
G1T571	Sulfotransferase	HS3ST3A1	−2.04	0.01
A0A5F9D6Z6	Myelin regulatory factor like	MYRFL	−2.05	<0.01
G1TAN8	Amine oxidase	AOC2	−2.06	<0.01
G1TNI4	Glutathione transferase	GSTM2	−2.08	<0.01
G1SPR7	Uncharacterized protein	​	−2.14	<0.01
G1STE2	Sulfotransferase	HS3ST1	−2.15	<0.01
A0A5F9C9K0	BMP binding endothelial regulator	BMPER	−2.16	<0.01
A0A5F9D9E9	Citrulline aspartate ligase	​	−2.20	<0.01
G1SJP8	Bone morphogenetic protein 6	BMP6	−2.24	<0.01
E1AWE3	Osteoprotegerin (fragment)	TNFRSF11B	−2.34	<0.01
B9DR19	C1q tumor necrosis factor alpha related protein 7 (fragment)	CTRP7	−2.47	<0.01
G1TUM4	Gremlin 1, DAN family BMP antagonist	GREM1	−2.56	<0.01
G1SSS1	Cartilage intermediate layer protein	CILP	−2.57	<0.01
D5FIT0	Lipoprotein lipase	LPL	−2.58	0.01
A0A5F9CDX1	ABI family member 3 binding protein	ABI3BP	−2.72	<0.01
Q27Q51	Protein Wnt	Wnt-11	−2.86	0.01
G1T4P1	Angiopoietin like 7	ANGPTL7	−3.40	<0.01

The threshold is set at Log_2_FC ≥ 2 and *p* < 0.05. Abbreviation: FC, fold change.

The original abundance of Mmp13 increased 3-fold in the MF group compared to that in the CON group (Figure 2G), and that of Col1a1 showed a 6-fold increase (Figure 2H), which was consistent with the abnormal ECM composition observed in the regenerated tissue. In addition, Tf abundance increased 6-fold in the MF-induced cartilage regeneration (Figure 2F), indicating iron deposition and iron toxicity underlying the abnormal ECM constitution and chondrocyte dysfunction. More than 80% of the circulating Fe^3+^ in mammals is contained in heme, the precursor of hemoglobin, which carries oxygen in the bloodstream (Chiang et al., 2021). In addition, heme is degraded by the constitutive microsomal rate-limiting heme-related enzyme Hmox2 under conditions of normal physiological intake (Singhabahu et al., 2023). Albumin (Alb) is the most abundant protein in the blood (Farshbaf et al., 2022). Herein, the expression levels of Hmox2 and Alb increased 3-fold and 8-fold, respectively, in the MF group compared to those in the CON group (Figures 2I,J), indicating enhanced heme degradation and a potential source of iron in insufficient cartilage regeneration.

### 3.3 Altered lipid metabolism in the regenerated cartilage

**FIGURE 3:**
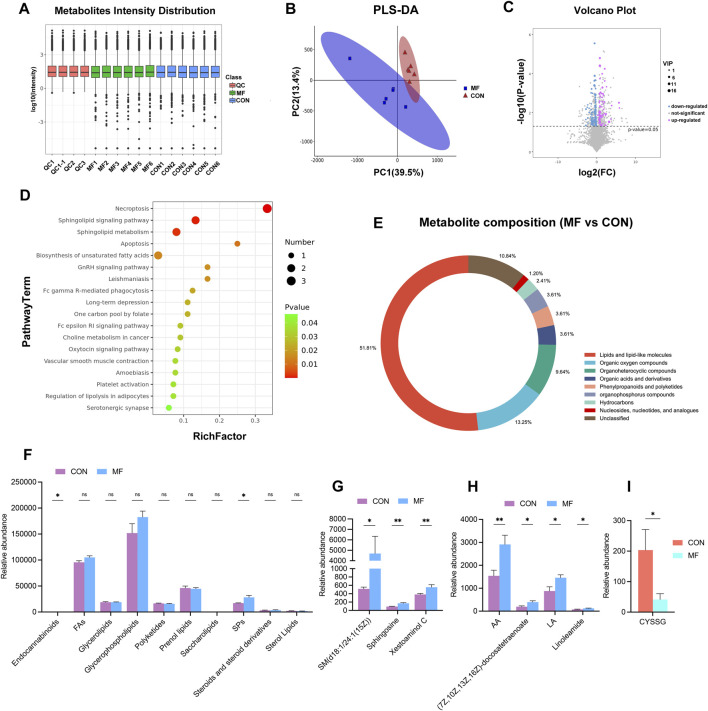
Metabolomic profile alterations in MF-induced cartilage regeneration. **(A)** Six MF cartilages and six normal cartilages are subjected to metabolome analysis. **(B)** PLS–DA reveals the distinct difference between the CON and MF groups. **(C)** Volcano plot reveals the DEMs at a threshold of *p* < 0.05, with the upregulated metabolites in red and downregulated metabolites in blue. **(D)** The KEGG enrichment of DEMs (*p* < 0.05). The bubble size represents the number of members detected in the pathway, and the color of the bubble represents the *p*-value. **(E)** The pie chart indicates the metabolic alteration at the metabolite species level. **(F)** The bar graph indicates the lipidomic alteration at the lipid species level. **(G)** Expressions of SPs in DEMs are revealed between the CON and MF groups. **(H)** Expressions of several FAs in DEMs are revealed between the CON and MF groups. **(I)** Expressions of CYSSG are revealed between the CON and MF groups. Data are presented as the mean ± SE (n = 6). *p < 0.05, **p < 0.01.

The *in situ* biochemical niche interacts with the behavior of newborn chondrocytes. To determine the MF-induced metabolic alteration, 83 DEMs were identified between the MF and CON groups (Figures 3A–C; Supplementary Table S4). The alterations in SP signaling and SP metabolism were highlighted by KEGG enrichment (Figure 3D). The lipids and lipid-like molecules accounted for half of the metabolic alteration in cartilage regeneration (Figure 3E). Specifically, the relative abundance of SPs significantly increased in the MF group compared to that in the CON group, while FAs showed an increasing trend (Figure 3F). Consistently, the contents of the specific SPs and FAs, such as SM (d18:1/24:1(15Z), sphingosine, arachidonic acid (AA), and linoelaidic acid (LA), were increased in the regenerated tissue (Figures 3G,H).

Glutathione (GSH) metabolism serves as a buffering system, scavenging the ROS and attenuating lipid oxidation (Thayyullathil et al., 2021). During detoxification of peroxides, GSH is oxidized to glutathione disulfide. L-cysteine-glutathione disulfide (CYSSG) is a GSH derivative comprised of the oxidized form of the free GSH tripeptide. In this study, although GSH was absent from the DEM list, the differentially expressed pattern of CYSSG was identified, showing an increase in MF-induced tissue (Figure 3I), which contributes to moderate oxidative stress in insufficient cartilage regeneration.

### 3.4 Iron-predisposed chondrotoxicity potentially alters sphingolipid flux

**FIGURE 4:**
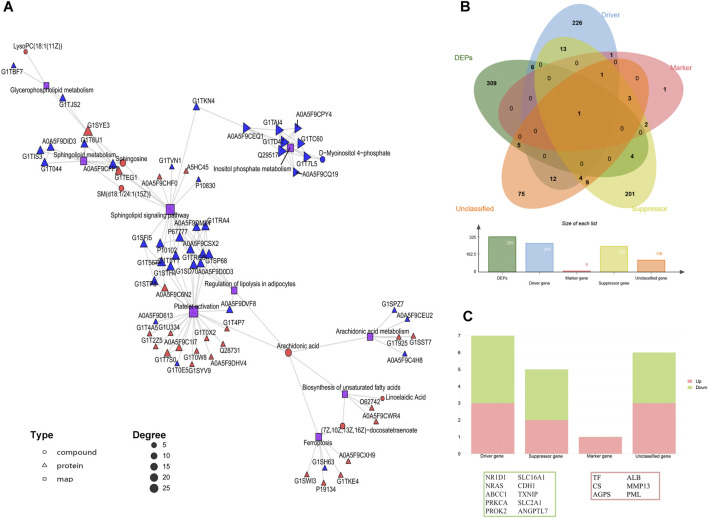
Integrated omics analysis elucidates a pre-ferroptotic state in MF-induced cartilage regeneration. **(A)** The KGML network highlights the ferroptosis-related biological processes. **(B)** DEPs are intersected with 621 ferroptosis-related genes from the FerrDb database. Four classes are shown in the Venn diagram: the ferroptosis driver is shown in blue, the marker is shown in pink, the suppressor is shown in yellow, and the unclassified gene is shown in purple. **(C)** Trends of the identified ferroptosis-related DEPs in each class (Supplementary Table S5).

By integrating the bulk information of proteins and metabolites, the biological processes, including glycerophospholipid metabolism, SP signaling, SP metabolism, platelet activation, and ferroptosis, among others, were detected (Figure 4A; Supplementary Figure S3). It indicates that iron toxicity potentially altered the SP flux, which underlies the insufficient cartilage regeneration and a ferroptosis-like event in the newborn chondrocytes. Specifically, the intersection of the 325 DEPs with the genes listed in the FerrDb (Liu et al., 2022; Zhu et al., 2022) identified 16 proteins that potentially mediate the iron-predisposed chondrotoxicity (Figures 4B,C; Supplementary Table S5).

### 3.5 Iron toxicity predisposes regenerating chondrocytes to a pre-ferroptotic state

**FIGURE 5:**
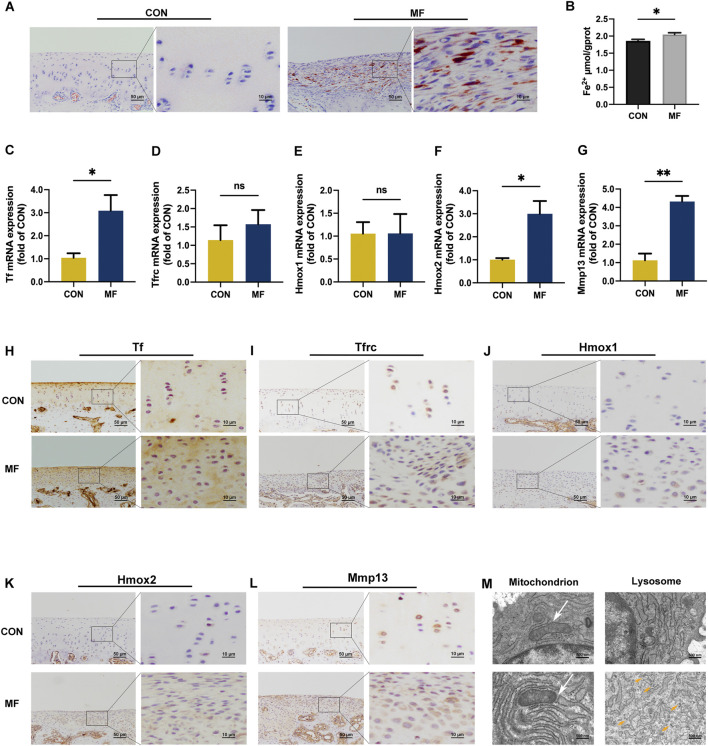
Iron deposits and ferroptotic pre-conditioning occur in insufficient cartilage regeneration. **(A)** DAB-enhanced Perls’ Prussian blue staining indicates excess Fe^3+^ deposits in MF-induced regenerated cartilage. **(B)** Quantitative analysis using the iron assay kit reveals a slight elevation in Fe^2+^ content in the regenerated tissue compared to that in intact cartilage (*n* = 3). **(C–G)** Relative mRNA expressions of Tf, Tfrc, Hmox1, Hmox2, and Mmp13 between the CON and MF groups (n = 3). Data are presented as the mean ± SE. ns indicates no significance (*p* > 0.05); *p < 0.05; **p < 0.01. (H–L) Immunohistochemical staining of Tf, Tfrc, Hmox1, Hmox2, and Mmp13. (M) TEM observations of mitochondrion (white arrow) and lysosome (orange arrow) in the chondrocytes.

Fe^3+^ deposits were detected beneath the superficial layer of the regenerated tissue, primarily within the extracellular matrix. In contrast, no corresponding signal was observed in intact cartilage (Figure 5A). The labile Fe^2+^ pool, which drives lipid peroxidation and mitochondrial dysfunction (Stockwell, 2022), showed a moderate increase in regenerated cartilage compared to that in intact tissue (Figure 5B).

Tfrc facilitates the internalization of Fe^3+^-loaded Tf (Stockwell, 2022). The mRNA expression of Tf was higher in the regenerated tissue than in the intact tissue (Figure 5C). Tf staining in the middle-to-deep layers is paralleled with the Fe^3+^ deposits in the regenerated tissue; that is, Tf-positive staining was detected on the chondrocyte membrane in the middle and deep layers of the regenerated tissue, but only in the thin superficial layer of the tissue under intact conditions (Figure 5H). Although the immune-positive staining of Tfrc increased with the elevated number of chondrocytes at the original defect site (Figure 5I), the relative mRNA expression of Tfrc was similar between the MF and CON groups (Figure 5D). This may be consistent with the extracellular distribution of Fe^3+^ in the regenerated tissue.

Hmox1, an inductive microsomal rate-limiting heme-related enzyme, responds to cellular stress and produces pro-oxidative Fe^2+^ (Singhabahu et al., 2023), and it showed comparable mRNA levels between the MF and CON groups (Figure 5E) despite increased immuno-positive staining associated with higher chondrocyte density in the defect site (Figure 5J). This pattern indicates that newborn chondrocytes experience oxidative stress, likely attributable to the elevated Fe^2+^ content in regenerated tissue. In contrast, both mRNA and protein expression of Hmox2, which functions in basal heme degradation (Singhabahu et al., 2023), were significantly upregulated in the MF group (Figures 5F,K), indicating the accumulation of excess heme following the microfracture procedure.

Mitochondria serve as both initiators and amplifiers of ferroptosis (Kondadi et al., 2020). Lysosomes function as iron-storage compartments and sites of SM catabolism (Thayyullathil et al., 2021; Pamarthi et al., 2022). Glycosphingolipids, a subclass of SPs, accumulate within lysosomes, forming lipofuscin granules that contain Fe^2+^. These granules promote lipid oxidation and can ultimately lead to plasma membrane rupture via ferroptosis (Tian et al., 2021). In newborn chondrocytes, mitochondria appeared shrunken and electron-dense, with reduced cristae formation, while lysosomes were notably increased in number (Figure 5M). This ultrastructural profile is indicative of oxidative stress overwhelming the GSH buffering system, which is characteristic of a pre-ferroptotic state (Kondadi et al., 2020). Furthermore, lysosomal catabolism of matrix proteins has been shown to suppress ferroptosis in cancer models (Armenta et al., 2022). Consistent with this, both mRNA expression and protein staining of Mmp13 were increased in regenerated cartilage (Figures 5G,L), indicating a parallel adaptive response in the repair tissue.

### 3.6 Chelating iron facilitates cartilage regeneration

**FIGURE 6:**
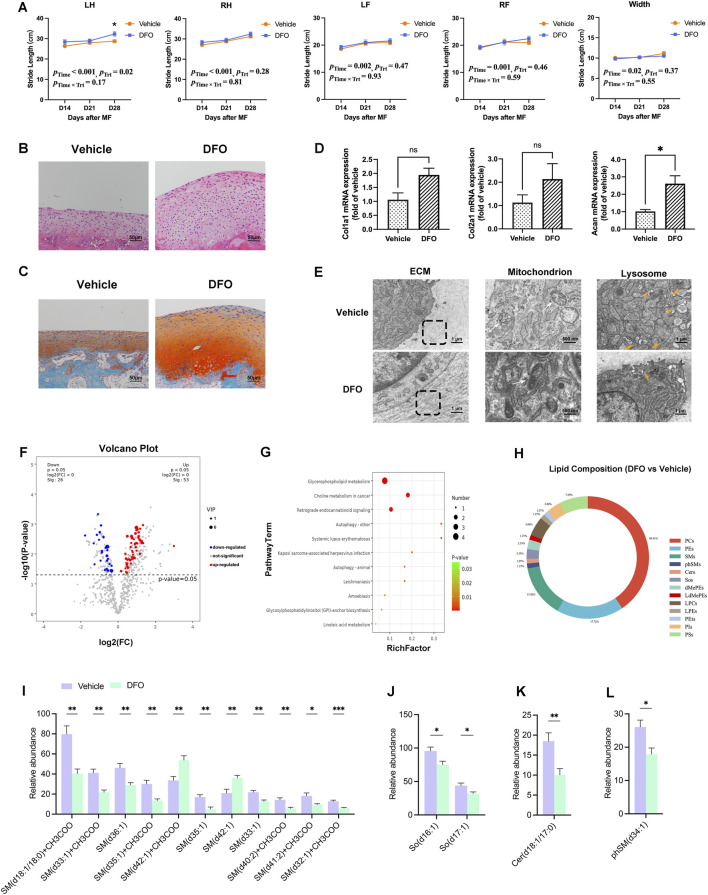
DFO improves MF outcomes. **(A)** Gait analysis between DFO and vehicle rabbits (n = 6). **(B)** H&E staining of the regenerated cartilages from the DFO and vehicle groups (n = 3). **(C)** SO/FG staining (n = 3). **(D)** Relative mRNA expressions of Col1a1, Col2a1, and Acan in the DFO and vehicle groups. Data are presented as the mean ± SE (n = 3). ns indicates no significance (*p* > 0.05); *p < 0.05. **(E)** TEM observations of ECM, mitochondria (white arrow), and lysosomes (orange arrow). The images are the representative result of three biological replicates. **(F)** The volcano plot indicates the DEMs identified by untargeted lipidomics (*p*-value <0.05, VIP >1), with upregulated DEMs shown in red, downregulated DEMs shown in blue, and non-significantly altered metabolites shown in gray. **(G)** KEGG enrichment of DEMs. **(H)** The pie chart reveals the alterations at the lipid species level. The differentially expressed patterns of each **(I)** SM, **(J)** SO, **(K)** Cer, and **(L)** phSM have been specified between the DFO and vehicle groups. Data are presented as the mean ± SE (n = 6). *p < 0.05, **p < 0.01, and ***p < 0.001.

To modify the ferroptotic state predisposed to the newborn chondrocytes, the improvement of the MF outcome was analyzed by chelating excess iron in cartilage regeneration. The joint mobility of MF limbs was improved by DFO injection (p_Time_ < 0.001, p_Trt_ = 0.02, p_Time × Trt_ = 0.17; Figure 6A). Particularly, on day 28 post-surgery, the LH stride length in the DFO group was longer than that in the vehicle group (p_LH_ = 0.02, Figure 6A).

The thickness and cellular regularity of the regenerated cartilage were facilitated (Figure 6B). The reddish-stained area was enlarged in SO/FG staining, and the reddish color was enhanced, indicating increased proteoglycan content in the regenerated tissue by DFO injection (Figure 6C). Moreover, the mRNA expression of Acan was significantly increased in the DFO group compared to the vehicle group, whereas the uptrends of Col1a1 and Col2a1 were observed without reaching statistical significance (Figure 6D). Moreover, DFO preserved the morphology of chondrocyte mitochondria (Figure 6E). Specifically, the mitochondria in the vehicle group were dense with poorly defined cristae, whereas in the DFO group, mitochondria were elongated with more prominent cristae. Additionally, DFO administration decreased the number of lysosomes and increased fibrous structures in the ECM (Figure 6E).

The lipidomic profile was modified by iron chelation. A total of 79 DEMs were identified (Figure 6F). The KEGG enrichment highlighted alterations in glycerophospholipid metabolism and LA metabolism (Figure 6G). Generally, phosphatidylcholines (PCs), phosphatidylethanolamines (PEs), and SMs accounted for 72.15% of the lipid metabolic alteration (Figure 6H). The reduced trend of each DEM belonging to SPs induced by DFO was delineated (Figures 6I–L), including SMs, sphingosines (SOs), Cers, and sphingomyelin phytosphingosines (phSMs). Given that iron toxicity triggers the alteration of SP signaling (Lee et al., 2012), the striking SP level was decreased by DFO as the excess iron in MF has been chelated.

### 3.7 Inhibiting iron-dependent oxidative stress facilitates cartilage regeneration

**FIGURE 7:**
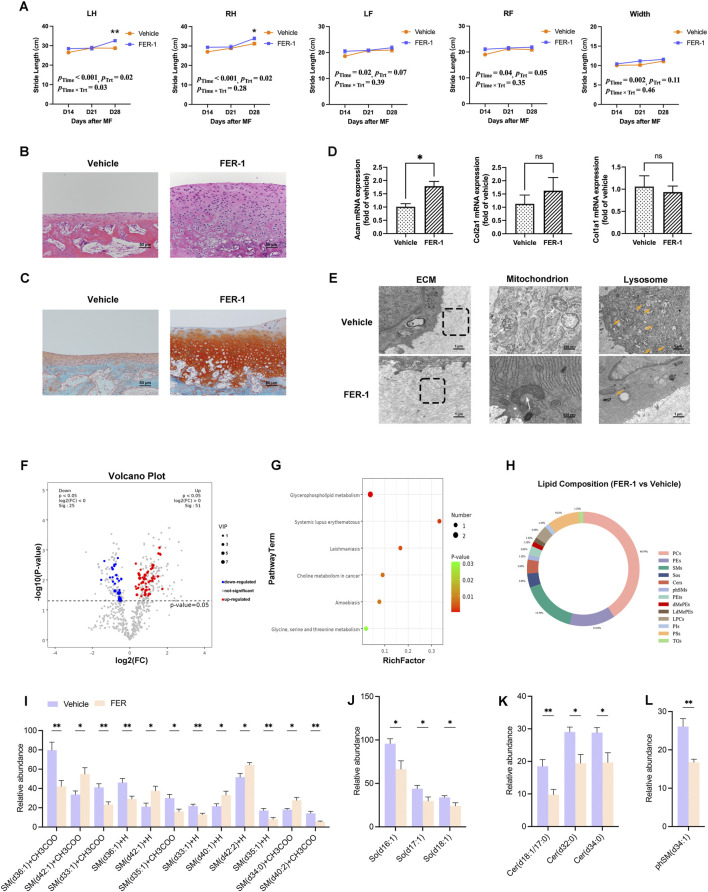
FER-1 improves MF outcomes. **(A)** Gait analysis between FER-1 and vehicle rabbits (n = 6). **(B)** H&E staining of the regenerated cartilages from the FER-1 and vehicle groups (n = 3). **(C)** SO/FG staining (n = 3). **(D)** Relative mRNA expressions of Col1a1, Col2a1, and Acan in the FER-1 and vehicle groups. **(E)** TEM observations of ECM (white arrow) and lysosomes (orange arrow). The images are the representative result of three biological replicates. **(F)** The volcano plot indicates the DEMs identified by untargeted lipidomics (*p*-value <0.05, VIP >1), with upregulated DEMs shown in red, downregulated ones in blue, and non-significantly altered metabolites in gray. **(G)** KEGG enrichment of DEMs. **(H)** The pie chart reveals the alterations at the lipid species level. The differentially expressed patterns of each **(I)** SM, **(J)** SO, **(K)** Cer, and **(L)** phSM have been specified between the FER-1 and vehicle groups. Data are presented as the mean ± SE (n = 6). *p < 0.05; **p < 0.01.

Because iron-dependent ROS accumulation mediates iron toxicity, the administration of antioxidants and ferroptosis inhibitors was proposed to rescue the predisposed ferroptotic state in newborn chondrocytes and improve MF outcomes. The joint mobility of the hindlimbs was improved by FER-1 injection (left: p_Time_ < 0.001, p_Trt_ = 0.02, and p_Time × Trt_ = 0.03; right: p_Time_ < 0.001, p_Trt_ = 0.02, and p_Time × Trt_ = 0.03; Figure 7A). The stride length of LH and RH on day 28th post-MF was higher in the FER-1 group than in the vehicle group (p_LH_ = 0.05, p_RH_ = 0.05; Figure 7A).

The thickness, cellular regularity, chondrocyte density, and proteoglycan content of regenerated tissue were improved via treatment with FER-1 (Figures 7B,C). Moreover, the mRNA expression of Acan was increased in FER-1-treated regenerated tissue compared to that with the vehicle (Figure 7D). For Col1a1 and Col2a1, upward trends were observed in the FER-1 group, although these did not reach statistical significance (Figure 7D). However, collagen fibers in the ECM were slim yet well-arranged in the FER-1 group, while those in the vehicle matrix were loose and disorganized (Figure 7E). The mitochondria became narrower, the cristae became longer and arranged, and the number of lysosomes was reduced compared to that in the vehicle group (Figure 7E).

The concentrations of 76 metabolites were modified by FER-1 (Figure 7F). Ferroptosis causes cell death by damaging the cell membranes, while glycerophospholipids are the major lipid components of the plasma membrane (Rocha et al., 2015). In the regenerated cartilage, glycerophospholipid metabolism was altered (Figure 7G). In general, PCs and SMs accounted for 56.58% of the lipidomic alterations (Figure 7H). In addition, the alterations were shown for each SP, including SMs, SOs, Cers, and phSMs (Figures 7I–K).

## 4 Discussion

Arthroscopic MF is a first-line treatment for small cartilage defects (Chiang et al., 2020), yet we propose that heme-derived iron establishes a high-iron microenvironment in the regenerative niche. Although iron toxicity may not immediately trigger full-scale ferroptosis, the resulting lipid peroxidation and lysosomal stress serve as upstream stimuli that prime the expression of apoptotic and necroptotic markers (Figure 3D). This process begins with extracellular Fe^3+^ deposition, which upregulates Tf expression. Under physiological conditions, most Fe^3+^ remains sequestered in the extracellular matrix, with only limited internalization via Tfrcs. Internalized Fe^3+^ is partly stored in enlarged lysosomes, which alter matrix composition through protein catabolism, and partly released via Hmox2-mediated heme degradation. Concurrently, the limited Hmox1 expression results in a modest increase in Fe^2+^, which generates ROS and imposes manageable yet persistent oxidative stress on newborn chondrocytes. Ultimately, extracellular Fe^3+^ accumulation induces a pre-ferroptotic state that undermines cartilage regeneration despite an increased chondrocyte presence.

When Tfrc levels increase—facilitating Fe^3+^ internalization—and ROS production exceeds the GSH buffering capacity, it triggers lysosomal membrane permeabilization and subsequent Fe^3+^ leakage. This cascade replenishes the labile Fe^2+^ pool via Hmox1 activity, initiating ferroptosis and widespread lipid peroxidation. The abundant SM are catabolized into Cers, which accumulate during ferroptosis and are required for erastin-induced ROS and lipid peroxide generation (Thayyullathil et al., 2021; Vu et al., 2022), ultimately leading to ferroptosis in regenerated chondrocytes. Notably, in primary OA, excess intracellular iron in chondrocytes has been shown to increase oxidative stress, initiate ferroptosis, and contribute to cartilage damage (Cai et al., 2021; Guo et al., 2022; Cao et al., 2024; Yang et al., 2024). This mechanism may also underlie the frequent diagnosis of secondary OA within 5–10 years following MF surgery (Goyal et al., 2014), which is potentially linked to the progressive internalization of Fe^3+^ from the ECM. Established ferroptosis inhibitors, such as DFO and FER-1, offer therapeutic promise; our findings support their potential to improve MF outcomes and delay the onset of secondary OA. In clinical settings, serum SPs are linked to early-stage OA (Eichner et al., 2025). Although the interplay between SP signaling and iron toxicity impairs cartilage regeneration, SP metabolism remains essential for bone remodeling (Qi et al., 2021). Metabolites such as phosphocholine, specific SMs, and PCs are present during early chondrogenesis and serve as lipid markers of chondrogenic differentiation (Rocha et al., 2015). Thus, while preserving essential lipid signaling, reducing iron toxicity should be prioritized in refining MF-based cartilage-repair strategies. Despite the comprehensive landscape provided by our multi-omics integration and the functional validation through pharmacological interventions (DFO and FER-1), we acknowledge that the current study is primarily correlative in nature. To further consolidate these mechanistic insights, future research utilizing conditional gene knockout models is warranted to definitively dissect the causal necessity and sufficiency of these specific metabolic regulators in the context of MF-induced cartilage regeneration.

Blood rich in free iron plays a dual role in cartilage repair: it supplies endogenous BMSCs to the defect site to promote regeneration in MF, yet it also deposits excess iron in the regenerative niche, ultimately undermining cartilage repair. This iron-mediated chondrocyte toxicity is exemplified in arthropathies associated with hemochromatosis and hemophilia (Schumacher, 1982; Gualtierotti et al., 2021). Hemochromatosis, a genetic disorder characterized by pathological iron accumulation, is primarily caused by mutations in the human homeostatic iron regulator gene HFE and, less frequently, in TFR2 or other genes (Schumacher, 1982). Arthritis is a major clinical manifestation of hemochromatosis, and studies indicate that iron toxicity can trigger irreversible catastrophic cascades in damaged cartilage explants, even after iron removal (Ferreira et al., 2021). Hemophilic arthropathy, the most common complication of hemophilia, results from recurrent joint bleeding that progressively destroys the articular lining, including the cartilage and synovium (Gualtierotti et al., 2021). It has been reported that iron overload induces hemophilic cartilage lesions by modulating the chondrocyte phenotype, degrading the cartilage ECM, and triggering chondrocyte apoptosis (Zheng et al., 2023). Given that Tf is expressed in the superficial layer of intact cartilage, regulated, low-level iron import via this canonical pathway may be essential for chondrocyte homeostasis. This indicates that physiological iron levels in synovial fluid are tightly controlled. Joint bleeding or hemarthrosis disrupts this balance, exposing chondrocytes to iron toxicity, while concomitant synovial inflammation accelerates cartilage breakdown—a mechanism likely underlying the permanent and irreversible joint damage seen in hemophilic arthropathy.

## 5 Conclusion

**FIGURE 8:**
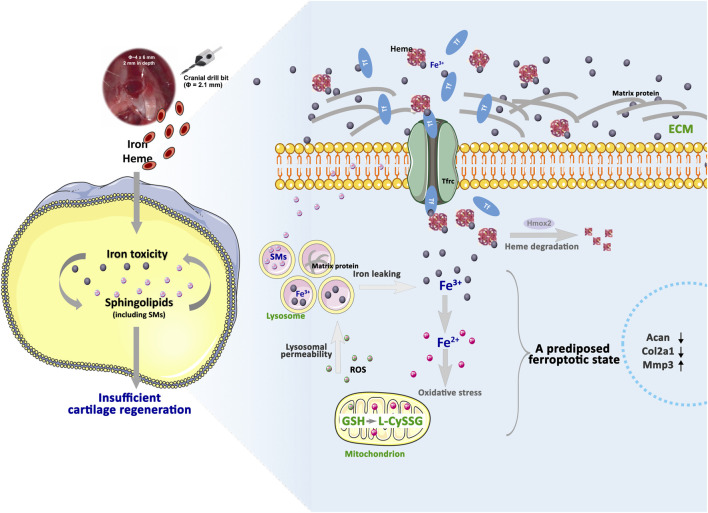
Iron toxicity mediated by SP signaling predisposes a ferroptotic condition to the newborn chondrocytes, which underlies insufficient cartilage regeneration in MF.

This study demonstrates that heme-derived iron released during MF establishes a pre-ferroptotic niche in newborn chondrocytes, characterized by extracellular Fe^3+^ deposition and elevated intracellular labile Fe^2+^ pools (Figure 8). This high-iron environment drives SP flux, which heightens cellular sensitivity to oxidative stress and compromises the production of key extracellular matrix components, notably aggrecan and type II collagen. Intra-articular administration of the iron chelator or the lipid ROS scavenger rescues this pre-ferroptotic state, thereby facilitating proteoglycan production and improving joint mobility. These results not only provide a novel mechanistic basis for improving MF-induced cartilage repair but also highlight iron-mediated SP signaling as a potential therapeutic target for a broader range of iron-overload-related joint disorders.

## Data Availability

The raw data supporting the conclusions of this article will be made available by the authors, without undue reservation.

## Glossary

MF: microfracture
ECM: extracellular matrix
Col1a1: type I collagen
Mmp3: matrix metalloproteinase 3
Col2a1: type II collagen
Acan: aggrecan
VIP: variable importance in projection
DEP: differentially expressed protein
DEM: differentially expressed metabolite
OA: osteoarthritis
Tf: transferrin
Hmox1: heme oxygenase 1
Hmox2: heme oxygenase 2
BMSC: bone marrow-derived mesenchymal stem cell
H&E: hematoxylin and eosin
SO/FG: safranin-O/fast green
PCA: principal component analysis
Alb: albumin
AA: arachidonic acid
LA: linoelaidic acid
SP: sphingolipid
SM: sphingomyelin
PUFA: polyunsaturated fatty acid
Tfrc: transferrin receptor
TEM: transmission electron microscopy
PC: phosphatidylcholine
PE: phosphatidylethanolamine
SO: sphingosine
Cer: ceramide
phSM: sphingomyelin phytosphingosine
Mmp13: matrix metalloproteinase 13

## References

[B1] Ajoolabady A. Aslkhodapasandhokmabad H. Libby P. Tuomilehto J. Lip G. Y. Penninger J. M. (2021). Ferritinophagy and ferroptosis in the management of metabolic diseases. Trends Endocrinol. & Metabolism 32, 444–462. 10.1016/j.tem.2021.04.010 34006412

[B2] Armenta D. A. Laqtom N. N. Alchemy G. Dong W. Morrow D. Poltorack C. D. (2022). Ferroptosis inhibition by lysosome-dependent catabolism of extracellular protein. Cell Chem. Biol. 29, 1588–1600. 10.1016/j.chembiol.2022.10.006 36306785 PMC9762237

[B3] Armiento A. Alini M. Stoddart M. (2019). Articular fibrocartilage - why does hyaline cartilage fail to repair? Adv. Drug Delivery Reviews 146, 289–305. 10.1016/j.addr.2018.12.015 30605736

[B4] Cai C. Hu W. Chu T. (2021). Interplay between iron overload and osteoarthritis: clinical significance and cellular mechanisms. Front. Cell Developmental Biology 9, 817104. 10.3389/fcell.2021.817104 35096841 PMC8795893

[B5] Cao S. Wei Y. Yue Y. Chen Y. Qian J. Wang D. (2024). Rosiglitazone retards the progression of iron overload-induced osteoarthritis by impeding chondrocyte ferroptosis. Iscience 27, 110526. 10.1016/j.isci.2024.110526 39224514 PMC11366908

[B6] Chen Y. Yang Z. Wang S. Ma Q. Li L. Wu X. (2023). Boosting ROS‐Mediated lysosomal membrane permeabilization for cancer ferroptosis therapy. Adv. Healthc. Mater. 12, 2202150. 10.1002/adhm.202202150 36408929

[B7] Chiang M. Kuo Y. Chen Y. (2020). Expanded mesenchymal stem cell transplantation following marrow stimulation is more effective than marrow stimulation alone in treatment of knee cartilage defect: a systematic review and meta-analysis. Orthop. & Traumatology, Surgery & Research OTSR 106, 977–983. 10.1016/j.otsr.2020.04.008 32536601

[B8] Chiang S.-K. Chen S.-E. Chang L.-C. (2021). The role of HO-1 and its crosstalk with oxidative stress in cancer cell survival. Cells 10, 2401. 10.3390/cells10092401 34572050 PMC8471703

[B9] Devitt B. Bell S. Webster K. Feller J. Whitehead T. (2017). Surgical treatments of cartilage defects of the knee: systematic review of randomised controlled trials. Knee 24, 508–517. 10.1016/j.knee.2016.12.002 28189406

[B10] Eichner G. Liebisch G. Hild C. Rickert M. Steinmeyer J. (2025). Serum phospholipids and sphingolipids are linked to early-stage osteoarthritis by lipidomic profiling. Arthritis Res. & Ther. 27, 69. 10.1186/s13075-025-03537-4 40165249 PMC11956431

[B11] Farshbaf M. Valizadeh H. Panahi Y. Fatahi Y. Chen M. Zarebkohan A. (2022). The impact of protein corona on the biological behavior of targeting nanomedicines. Int. J. Pharm. 614, 121458. 10.1016/j.ijpharm.2022.121458 35017025

[B12] Ferreira A. Duarte T. Marques S. Costa P. Neves S. Dos Santos T. (2021). Iron triggers the early stages of cartilage degeneration *in vitro:* the role of articular chondrocytes. Osteoarthr. Cartil. Open 3, 100145. 10.1016/j.ocarto.2021.100145 36474980 PMC9718170

[B13] Gomoll A. (2012). Microfracture and augments. Journal Knee Surgery 25, 9–15. 10.1055/s-0031-1299654 22624242

[B14] Goyal D. Keyhani S. Goyal A. Lee E. Hui J. Vaziri A. (2014). Evidence-based status of osteochondral cylinder transfer techniques: a systematic review of level I and II studies. Assoc. N. Am. Int. Arthrosc. 30, 497–505. 10.1016/j.arthro.2013.12.023 24680310

[B15] Gualtierotti R. Solimeno L. P. Peyvandi F. (2021). Hemophilic arthropathy: current knowledge and future perspectives. J. Thrombosis Haemostasis 19, 2112–2121. 10.1111/jth.15444 34197690 PMC8456897

[B16] Guo Z. Lin J. Sun K. Guo J. Yao X. Wang G. (2022). Deferoxamine alleviates osteoarthritis by inhibiting chondrocyte ferroptosis and activating the Nrf2 pathway. Front. Pharmacology 13, 791376. 10.3389/fphar.2022.791376 35359876 PMC8964096

[B17] Isidori A. Borin L. Elli E. Latagliata R. Martino B. Palumbo G. (2018). Iron toxicity–its effect on the bone marrow. Blood Rev. 32, 473–479. 10.1016/j.blre.2018.04.004 29699840

[B18] Kim J.-H. Lee G. Won Y. Lee M. Kwak J.-S. Chun C.-H. (2015). Matrix cross-linking-mediated mechanotransduction promotes posttraumatic osteoarthritis, Proc. Natl. Acad. Sci. U S A. 112 **,** 9424–9429. 10.1073/pnas.1505700112 26170306 PMC4522801

[B19] Kondadi A. Anand R. Reichert A. (2020). Cristae membrane dynamics - a paradigm change. Trends Cell Biology 30, 923–936. 10.1016/j.tcb.2020.08.008 32978040

[B20] Lee Y.-J. Huang X. Kropat J. Henras A. Merchant S. S. Dickson R. C. (2012). Sphingolipid signaling mediates iron toxicity. Cell Metab. 16, 90–96. 10.1016/j.cmet.2012.06.004 22768841 PMC3653578

[B21] Leong D. J. Gu X. I. Li Y. Lee J. Y. Laudier D. M. Majeska R. J. (2010). Matrix metalloproteinase-3 in articular cartilage is upregulated by joint immobilization and suppressed by passive joint motion. Matrix Biol. 29, 420–426. 10.1016/j.matbio.2010.02.004 20153826 PMC2902573

[B22] Li M. Yin H. Yan Z. Li H. Wu J. Wang Y. (2022). The immune microenvironment in cartilage injury and repair. Acta Biomater. 140, 23–42. 10.1016/j.actbio.2021.12.006 34896634

[B23] Liu H. Deng Z. Yu B. Liu H. Yang Z. Zeng A. (2022). Identification of SLC3A2 as a potential therapeutic target of osteoarthritis involved in ferroptosis by integrating bioinformatics, clinical factors and experiments. Cells 11, 3430. 10.3390/cells11213430 36359826 PMC9657506

[B24] Liu J. Jing W. Wang T. Hu Z. Lu H. (2023). Functional metabolomics revealed the dual-activation of cAMP-AMP axis is a novel therapeutic target of pancreatic cancer. Pharmacol. Res. 187, 106554. 10.1016/j.phrs.2022.106554 36379357

[B25] Lv Z. Han J. Li J. Guo H. Fei Y. Sun Z. (2022). Single cell RNA-seq analysis identifies ferroptotic chondrocyte cluster and reveals TRPV1 as an anti-ferroptotic target in osteoarthritis. EBioMedicine 84, 104258. 10.1016/j.ebiom.2022.104258 36137413 PMC9494174

[B26] Miao Y. Chen Y. Xue F. Liu K. Zhu B. Gao J. (2022). Contribution of ferroptosis and GPX4's dual functions to osteoarthritis progression. EBioMedicine 76, 103847. 10.1016/j.ebiom.2022.103847 35101656 PMC8822178

[B27] Murphy M. Koepke L. Lopez M. Tong X. Ambrosi T. Gulati G. (2020). Articular cartilage regeneration by activated skeletal stem cells. Nat. Medicine 26, 1583–1592. 10.1038/s41591-020-1013-2 32807933 PMC7704061

[B28] Pamarthi S. H. H. Fefelova N. Gwathmey J. K. Sadoshima J. Xie L.-H. (2022). Lysosomal regulation of calcium and iron homeostasis and ferroptosis in mouse cardiomyocytes. Circulation 146, A14952. 10.1161/circ.146.suppl_1.14952

[B29] Peng H. Zhang Y. Ren Z. Wei Z. Chen R. Zhang Y. (2022). Cartilaginous metabolomics reveals the biochemical-niche fate control of bone marrow-derived stem cells. Cells 11, 2951. 10.3390/cells11192951 36230915 PMC9562901

[B30] Qi T. Li L. Weidong T. (2021). The role of sphingolipid metabolism in bone remodeling. Front. Cell Dev. Biol. 9, 752540. 10.3389/fcell.2021.752540 34912800 PMC8666436

[B31] Ripmeester E. Caron M. Van Den Akker G. Steijns J. Surtel D. Cremers A. (2021). BMP7 reduces the fibrocartilage chondrocyte phenotype through MMP2. Osteoarthr. Cartil. 29, S137. 10.1016/j.joca.2021.02.192 PMC8490443 34608249

[B32] Rocha B. Cillero‐Pastor B. Eijkel G. Bruinen A. L. Ruiz‐Romero C. Heeren R. M. (2015). Characterization of lipidic markers of chondrogenic differentiation using mass spectrometry imaging. Proteomics 15, 702–713. 10.1002/pmic.201400260 25346268

[B33] Ru Q. Li Y. Xie W. Ding Y. Chen L. Xu G. (2023). Fighting age-related orthopedic diseases: focusing on ferroptosis. Bone Res. 11, 12. 10.1038/s41413-023-00247-y 36854703 PMC9975200

[B34] Schumacher H. (1982). Articular cartilage in the degenerative arthropathy of hemochromatosis. Arthritis Rheumatism 25, 1460–1468. 10.1002/art.1780251212 7150378

[B35] Singhabahu R. Gamage S. M. K. Gopalan V. (2023). Pathological significance of Heme Oxygenase-1 as a potential tumor promoter in heme-induced colorectal carcinogenesis. Cancer Pathogenesis Ther. 2 (2), 65–73. 10.1016/j.cpt.2023.04.001 PMC11002664 38601482

[B36] Stockwell B. R. (2022). Ferroptosis turns 10: emerging mechanisms, physiological functions, and therapeutic applications. Cell 185, 2401–2421. 10.1016/j.cell.2022.06.003 35803244 PMC9273022

[B37] Thayyullathil F. Cheratta A. Alakkal A. Subburayan K. Pallichankandy S. Hannun Y. (2021). Acid sphingomyelinase-dependent autophagic degradation of GPX4 is critical for the execution of ferroptosis. Cell Death & Disease 12, 26. 10.1038/s41419-020-03297-w 33414455 PMC7791123

[B38] Tian R. Abarientos A. Hong J. Hashemi S. H. Yan R. Dräger N. (2021). Genome-wide CRISPRi/a screens in human neurons link lysosomal failure to ferroptosis. Nat. Neuroscience 24, 1020–1034. 10.1038/s41593-021-00862-0 34031600 PMC8254803

[B39] Valiyaveettil M. Mort J. Mcdevitt C. (2005). The concentration, gene expression, and spatial distribution of aggrecan in canine articular cartilage, meniscus, and anterior and posterior cruciate ligaments: a new molecular distinction between hyaline cartilage and fibrocartilage in the knee joint. Connect. Tissue Research 46, 83–91. 10.1080/03008200590954113 16019418

[B40] Vu N. T. Kim M. Stephenson D. J. Macknight H. P. Chalfant C. E. (2022). Ceramide kinase inhibition drives ferroptosis and sensitivity to cisplatin in mutant KRAS lung cancer by dysregulating VDAC-mediated mitochondria function. Mol. Cancer Res. 20, 1429–1442. 10.1158/1541-7786.MCR-22-0085 35560154 PMC9444881

[B41] Xu S. Lin Y. Zhao X. Liang Z. Hu Y. Chen Z. (2023). NIR triggered photocatalytic and photothermal bifunctional MOF nanozyme using for improving osteoarthritis microenvironment by repairing injured chondrocytes of mitochondria. Chem. Eng. J. 468, 143826. 10.1016/j.cej.2023.143826

[B42] Yang J. Chen D. He Q. Chen B. Pan Z. Zhang G. (2024). Arctiin alleviates knee osteoarthritis by suppressing chondrocyte oxidative stress induced by accumulated iron via AKT/NRF2/HO-1 signaling pathway. Sci. Rep. 14, 31935. 10.1038/s41598-024-83383-7 39738432 PMC11685860

[B43] Zhang F. Chen Z. Wu D. Tian L. Chen Q. Ye Y. (2021). Recombinant human GLP-1 beinaglutide regulates lipid metabolism of adipose tissues in diet-induced obese mice. iScience 24, 103382. 10.1016/j.isci.2021.103382 34841227 PMC8605346

[B44] Zheng L. Yao Y. Luo D. Han Z. Zhang X. Pang N. (2023). Iron regulates chondrocyte phenotype in haemophilic cartilage through the PTEN/PI3 K/AKT/FOXO1 pathway. Hematology 28, 2240585. 10.1080/16078454.2023.2240585 37493406

[B45] Zhu H. Tan J. Wang Z. Wu Z. Zhou W. Zhang Z. (2022). Bioinformatics analysis constructs potential ferroptosis-related ceRNA network involved in the formation of intracranial aneurysm. Front. Cellular Neuroscience 16, 1016682. 10.3389/fncel.2022.1016682 36313616 PMC9612944

