# Supplementary figures and images for: Iron toxicity undermines microfracture-induced cartilage regeneration by predisposing a pre-ferroptotic niche
Source: Front Cell Dev Biol. 2026 Mar 11;14:1784707. doi: 10.3389/fcell.2026.1784707 (PMC13013296; doi:10.3389/fcell.2026.1784707)

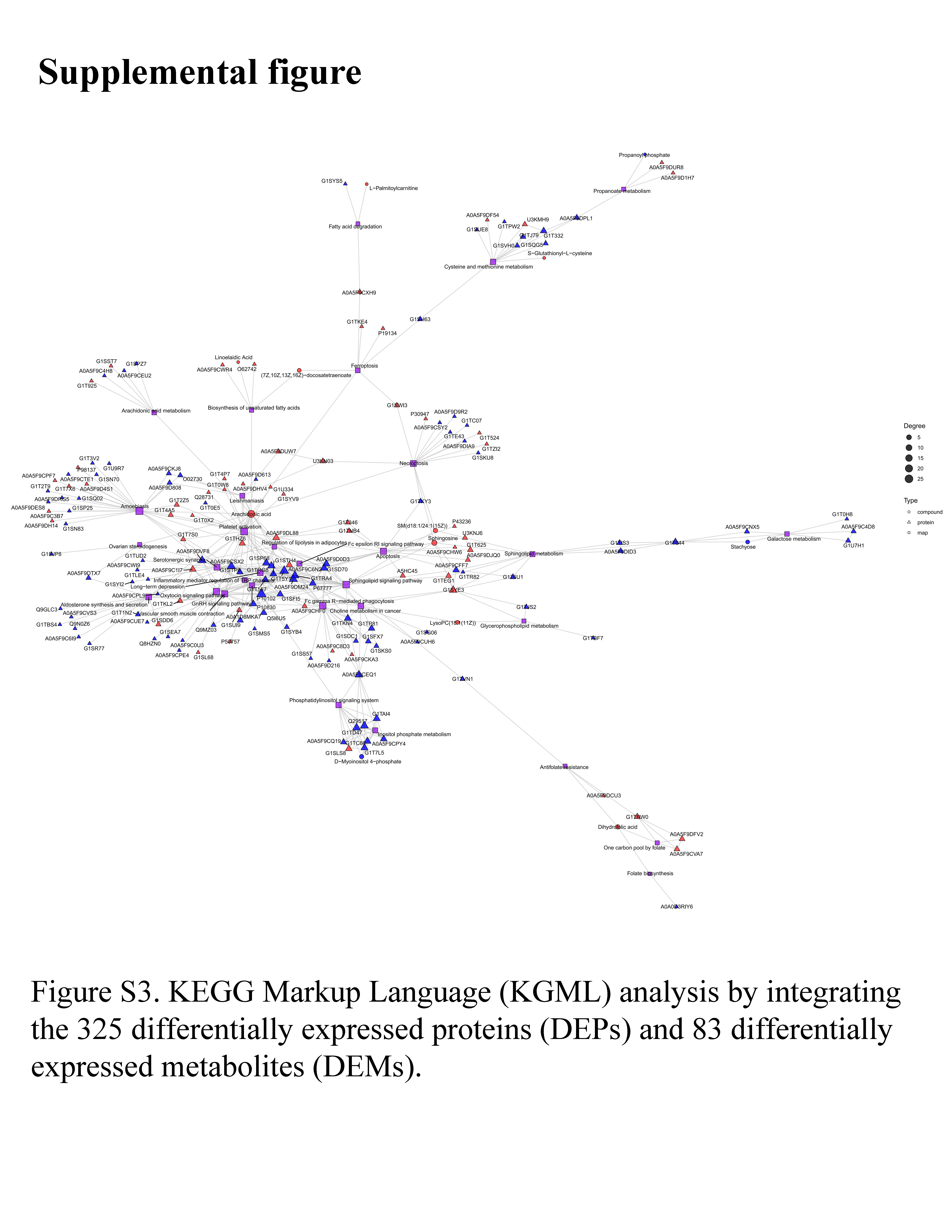

Supplement: Supplementary file 3 [file Image3.tif]

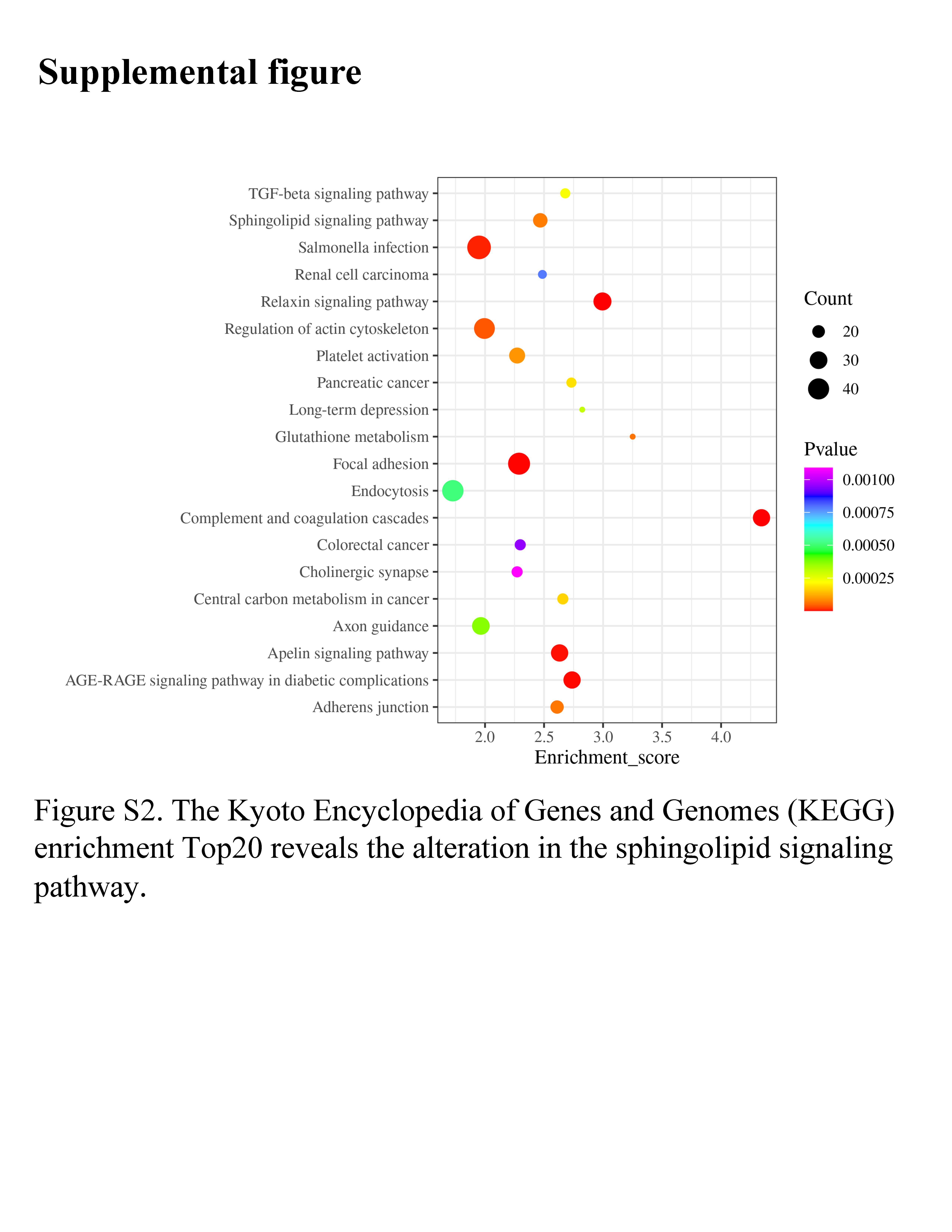

Supplement: Supplementary file 4 [file Image2.tif]

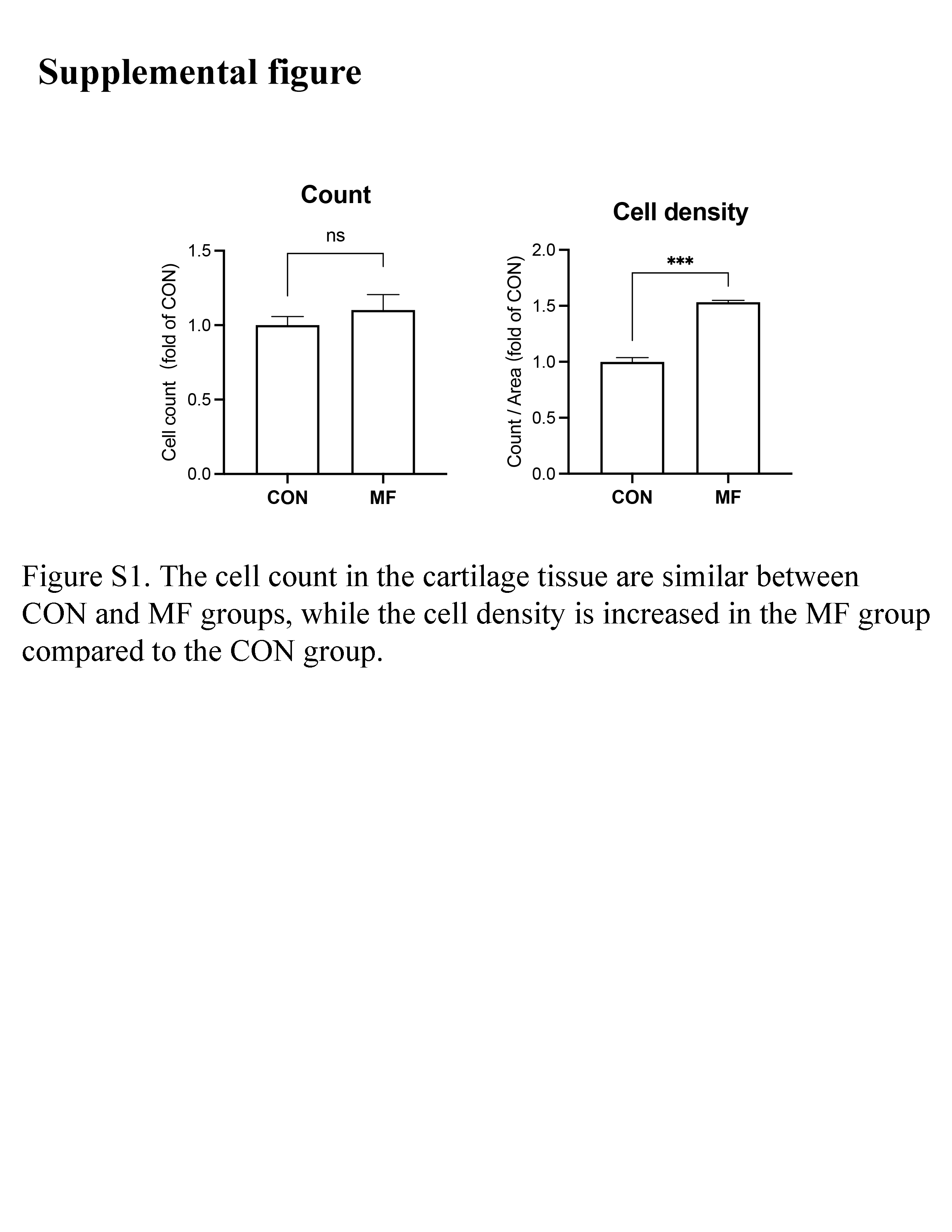

Supplement: Supplementary file 5 [file Image1.tif]
